# An integrative OSCE methodology for enhancing the traditional OSCE program at Taipei medical university ospital - a feasibility study

**DOI:** 10.1186/1472-6920-13-102

**Published:** 2013-07-26

**Authors:** Che-Wei Lin, Daniel L Clinciu, Mark H Swartz, Chien-Chih Wu, Gi-Shih Lien, Cho-Yu Chan, Fei-Peng Lee, Yu-Chuan Li

**Affiliations:** 1 Graduate Institute of Medical Informatics, Taipei Medical University, Taipei, Taiwan; 2 Department of Research and Education, Wan Fang Hospital, Taipei Medical University, Taipei, Taiwan; 3 Institute of Translational Medicine, Taipei Medical University, Taipei, Taiwan; 4 Department of International Trade, Feng Chia University, Taichung, Taiwan; 5 SUNY Downstate College of Medicine & C3NY, Clinical Competence Center of New York, New York, USA; 6 Department of Research and Education, Taipei Medical University Hospital, Taipei, Taiwan; 7 Department of Internal Medicine, Wan Fang Hospital, Taipei Medical University, Taipei, Taiwan; 8 Department of Otolaryngology, Wanfang Hospital, Taipei, Taiwan; 9 Department of Dermatology, Wanfang Hospital, Taipei, Taiwan

**Keywords:** Medical education, iOSCE, OSCE, Standardized patient, Virtual patient

## Abstract

**Background:**

Continuous development and use of new technologies and methodologies are key features in improving the learning, performance, and skills of medical students and students of all health care professions. Although significant improvements in teaching methodologies have been made in all areas of medicine and health care, studies reveal that students in many areas of health care taking an objective structured clinical examination (OSCE) express difficulties. Thus, this study was planned as a feasibility study to assess the educational effectiveness of an integrated objective structured clinical examination (iOSCE) using both standardized patients and virtual patients.

**Methods:**

Thirty (30) medical students in their first year of internship at Taipei Medical University volunteered to be part of a feasibility study for demonstrating the concept of iOSCE. They divided themselves into five groups of six students each and were requested to evaluate two cases: 1) a patient with abdominal pain and 2) a patient with headache using a combination of a standardized patient and a virtual patient. For each of the two cases, five stations were designed in which students were given ten minutes per station leading to a final diagnosis and concluded with a debriefing. The five stations were:

• Station 1) Interacting with the standardized patient.

• Station 2) Writing the patient note and developing a differential diagnosis.

• Station 3) Selecting appropriate laboratory and imaging studies.

• Station 4) Making a final diagnosis and stating the evidence for it.

• Station 5) Having the debriefing.

Each group of 6 students was assigned 2 hours per day for each case. All participants completed a survey regarding the usefulness and efficiency of the iOSCE.

**Results:**

All medical students (30/30; 100%) found the iOSCE program to be very satisfactory, and all expressed that they would like to have further iOSCE experiences if given the opportunity. In terms of ease and helpfulness, the students rated the program an average of 4.4 for the 1st case (abdominal pain) and 4.5 for the 2nd case (headache) on a scale of 1–5, with 5 being the highest and 1 being the lowest score.

**Conclusions:**

The participants felt that the iOSCE program can offer certain advantages over the traditional OSCE with the SP alone. They cited that the iOSCE provided improved clarity of what was being assessed as well as providing an opportunity to improve their diagnostic reasoning.

## Background

Continuous development of new, more sophisticated technologies and methodologies are key features in improving the clinical skills taught to medical students and other health care professionals. Medical colleges, schools of medicine, and other health care educational programs have traditionally assessed students’ performance using a combination of multiple-choice and essay questions. However, these methods of assessment may not adequately evaluate mastery of essential clinical skills and measure cognitive learning in clinical settings [1]. Furthermore, clinical faculty members often see a disparity between performance of high achievers in the classroom and in clinical settings [2,3]. This inconsistency may stem from differences in testing for memorization of information and clinical application of knowledge. Therefore, the use of performance-based assessment methods, such as the OSCE in health care education is of fundamental importance [4-11]. The OSCE has been used in evaluating clinical competence in health professions education around the world since Harden and Gleeson first described the concept in 1979 [12-14]. A Consensus Conference of the Association of American Medical Colleges (AAMC) in 1993 laid the groundwork for SP development in medical school education for teaching and assessing clinical skills [15].

At Taipei Medical University, we use SPs and VPs to create a new way of teaching and assessing the skills of medical students. Unlike the traditional OSCE where 10–12 stations are used, each having a different scenario or mission, iOSCE has five stations utilizing only one scenario or mission. The benefit of this procedure is for students to go through a more detailed and comprehensive problem solving. Students can also choose any information in the process without any given hints as opposed to OSCE where they have only paper available (e.g. x-rays chosen but no x-rays are available).

Since new technology and modern medical equipment has been constantly brought into providing medical and health care, strategies for teaching and evaluation in health care education must change as well [16-18]. This is also in tandem with the philosophy and practice of medical care which puts more emphasis on experiential training than didactic learning. Because more emphasis is being placed on the experiential aspect of training, more effective and more accurate evaluation of students’ performance is done in these practice settings [1,19]. However, the complexities of competencies tested at different OSCE stations may vary significantly and the clarity of instructions given to examinees, as well as the perceived degree of learning needed to achieve the competency being tested/evaluated, may also differ from one OSCE station to another [20-24]. Such wide variations may influence the validity and reliability of the overall examination. Another technique for assessing clinical skills is the mini-CEX. This is a direct observation tool for assessing medical interviewing skills, physical examination skills, humanistic qualities, professionalism, clinical judgment, counseling, organization, efficiency, and overall clinical competence. It is a 10–20 minute direct observation assessment or “snapshot” of a trainee-patient interaction. The faculty is encouraged to perform at least one per clinical rotation. To be most useful, faculty should provide timely and specific feedback to the trainee after each assessment of a trainee-patient encounter. The mini-CEX is a valuable tool, but is extremely faculty intensive.

The use of the VP in an OSCE-based exam has been piloted elsewhere with success [25-27]. Following such principles, this study aimed at enabling candidates to show how he/she could integrate the information obtained from the SP with the proper diagnostic blood and imagine studies obtained from the VP in order to develop a realistic differential diagnosis and plan. Thus, the current feasibility study aims to improve the students’ clinical skills as well as their perceptions of the OSCE stations’ effectiveness in evaluating competencies without substantial investments in cost and training time for faculty and students.

## Methods

This feasibility study was conducted at Taipei Medical University using the facilities of the first year medical education internship program.

### Ethics approval

The Department Chair of the Clinical Research Ethics Committee at Taipei Medical Hospital approved the study and confirmed that the study conformed to all applicable guidelines and that ethical matters were dealt with accordingly. An information sheet and a consent form were given to all participating students in the study. All students participating in the study had been given an information session explaining the project and signed the form of consent prior to this study.

### Integrated objective structured clinical examination (iOSCE) methodology

iOSCE is a new methodology at Taipei Medical University which combines the currently used OSCE methodology with an integrated, informative, investigative and innovative approach by using both virtual and standardized patients. The iOSCE program is used to develop a scenario (e.g., abdominal pain, back pain, headache) using an SP and creating the dialogue between the trainee and SP from the Virtual Patient System (DxR)© data.

A standardized patient (SP) is a person simulating the signs and symptoms of disease and is capable of providing feedback to the candidate on clinical skills of history-taking, physical examination, and interpersonal relationships. The term virtual patient (VP) is used to describe interactive computer simulations that are used in health care education. The VP is used for candidates to work through problems and situations that occur commonly in health care settings. The VP provides a consistent interactive and dynamic approach that promotes clinical reasoning and in-depth exploration of medical content.

### Participants

Thirty medical students in their first year of internship at Taipei Medical University volunteered to be part of the in a feasibility study for demonstrating the concept of iOSCE. They divided themselves into five (5) groups of six6 students each and were requested to evaluate two cases:

1) a patient with abdominal pain and 2) a patient with headache using a combination of a standardized patient and a virtual patient.

### Materials and procedures

**Figure 1:**
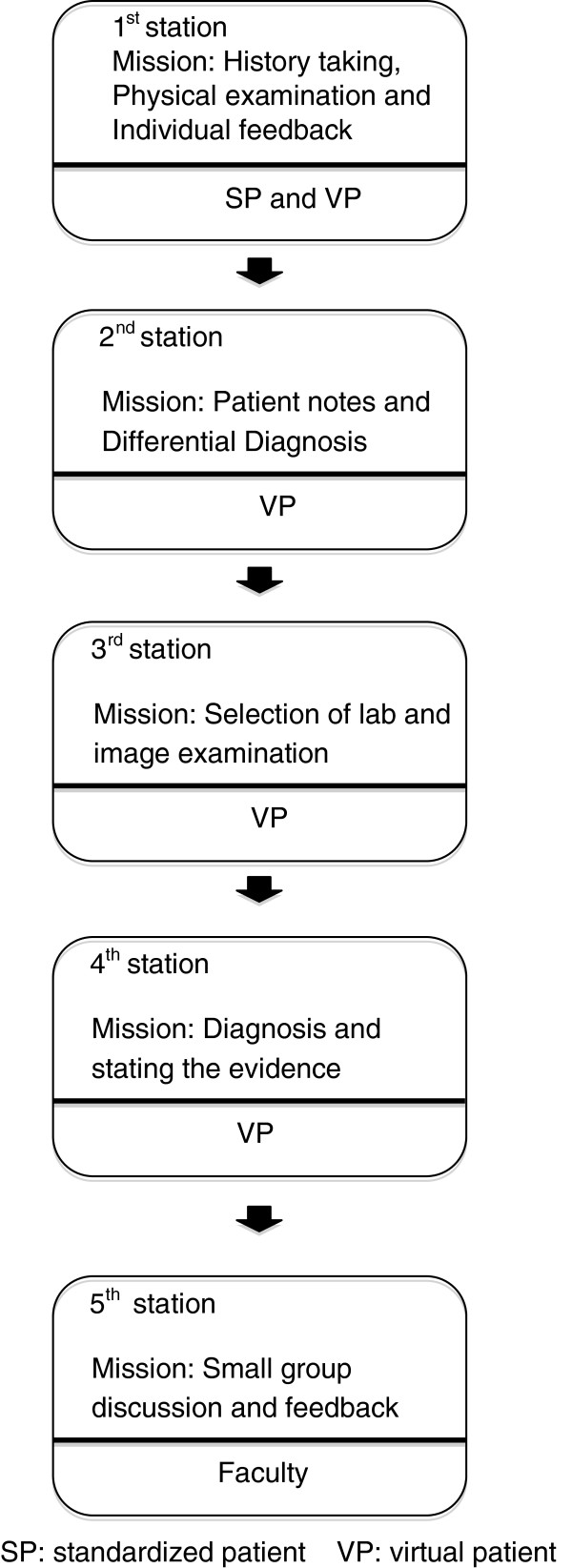
The flow chart of the iOSCE methodology.

To achieve a comprehensive and formative assessment of our students’ clinical competencies, this assessment model is designed as a short OSCE (using one specific scenario for a series of test sessions) that contains five integrated components set up at different stations (Figure 1). After providing a brief orientation to the examinees, the examinees went through the following stations to receive assessment of specific clinical skills as seen in Figure 1:

• Station 1 was planned for assessing the competencies of history taking and physical examination by using an SP.

• Station 2 was designed for assessing the ability to develop a differential diagnosis from the information obtained from previous station by using DxR Clinician© system differential diagnosis page.

• Station 3 was aimed at assessing the examinee’s ability to select appropriate laboratory and imaging tests and to interpret them using the DxR Clinician©.

• Station 4 assessed the examinee’s ability to establish a diagnosis by using DxR Clinician©.

• Station 5 provided feedback to the examinees by a physician tutor in a small group.

By combining the data from the VP and the SP, this approach gives students the opportunity to experience the emotions of a real patient, the ability to interact and interview a live patient and at the same time the examinee can decide on a workup and establish a differential diagnosis. This may improve both the method of assessing trainees and the venue for students to reflect and learn from their assessment. Through the iOSCE program, medical students have a chance to review themselves and improve their skills of critical reflection during clinical practice examinations.

## Results

Thirty medical students in their first year of internship participated in the iOSCE development. One hundred percent (30/30) of students in Case 1 and Case 2 stated that iOSCE was helpful while all expressed that they will attend again if given the opportunity. The satisfaction level of participants was an average of 4.4 for the 1st case (abdominal pain) and 4.5 on a scale of 5 (Table 1). The highest scores were obtained in the following four categories where the score in each category was 4.4 or above: 1) closest to a clinical setting (4.6 for case 1; 4.6 for case 2); 2) helped improve trainee’s clinic skills (4.6 for both cases); 3) iOSCE was helpful (4.5 for case 1 Table 1; 4.7 for case 2, Table 2); 4) participant will attend again if given the opportunity (4.8 for both cases). The only category that scored lower was the one emphasizing the difficulty felt by participants (3.6 for case 1; 3.9 for case 2).

**Table 1:** The participants’ rating of iOSCE for case 1 (abdominal pain)

**Rating scale (1–5)**	**Clinically relevant**	**Improving clinical skills**	**Helpful**	**Difficult**	**Will attend again**
Strongly agree (5)	18	19	17	4	23
Agree (4)	11	10	12	12	7
Somewhat agree (3)	1	1	1	12	0
Disagree (2)	0	0	0	0	0
Strongly disagree (1)	0	0	0	0	0
Average score	4.6	4.6	4.5	3.4	4.8

**Table 2:** The participants’ rating of iOSCE for case 2 (headache)

**Rating scale (1–5)**	**Clinically relevant**	**Improving clinical skills**	**Helpful**	**Difficult**	**Will attend again**
Strongly agree (5)	18	18	21	8	23
Agree (4)	12	11	9	13	7
Somewhat agree (3)	0	1	0	7	0
Disagree (2)	0	0	0	0	0
Strongly disagree (1)	0	0	0	0	0
Average score	4.6	4.6	4.7	3.9	4.8

## Discussion

From the students’ opinions, we concluded that the iOSCE training methodology provides a less stressful and a more helpful assessing and learning experience than a single SP or VP experience. The students indicated that they would like to participate again. The experience is quite comparable to that of a clinic setting. They expressed that the iOSCE allows them to “take charge” of the situation and their patient whereas this was not possible during the previous traditional OSCE setup [14].

The faculties have the responsibility to design and create student-centered curriculum within the current education trend. Implementing the iOSCE program at Taipei Medical Hospital is not only an improved assessment instrument for medical students, but it also serves as a new and improved teaching method through the integration of medical informatics, investigative and innovative methodologies embedded within OSCE. The feedback from students has encouraged the faculty to organize a new and complete iOSCE curriculum.

**Figure 2:**
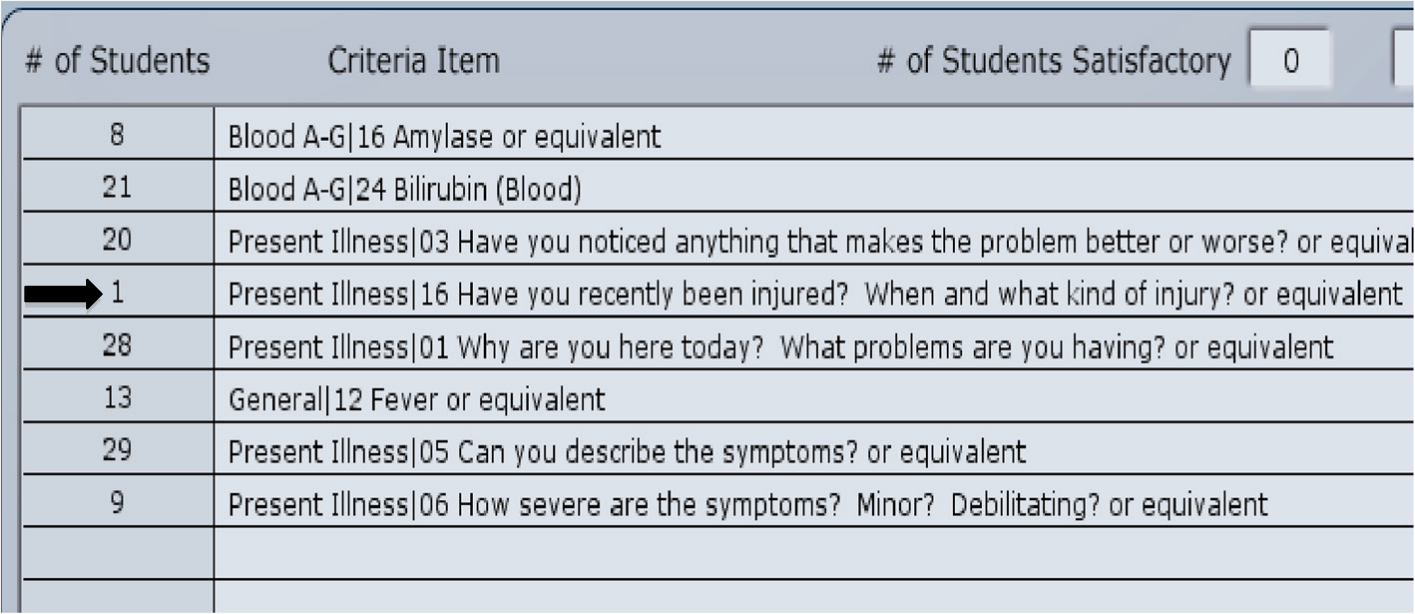
**The statistics table from the Utility Record.** Using the Utility Record the faculty and trainers can see the performance of each student and use it as feedback when training or teaching.

iOSCE allows the faculty to pinpoint student deficiencies from the statistics function of the virtual patient system. Since we record every activity of students, we can trace students’ thought processes, and use the statistics and data for analysis and teaching purposes. For example, in Case 1 (abdominal pain), we found that there was only one out of 30 students who asked “Have you had an abdominal injury recently?” (Figure 2). This suggests that most students didn’t even think that injury can be a cause for abdominal pain. Thus, the teacher can obtain valuable information on what the students have missed or what they forgot to do or ask based on the recordings from each station.

The combination of SP and VP used in iOSCE allows a medical student/trainee to write a plan of action and obtain the information about what kinds of tests the patient may need based on both interaction with the SP and the data available from DxR© Clinician. In addition, the use of debriefing in iOSCE also enhances learning by increasing confidence and reducing embarrassment of students in case they missed something, performed poorly during rotations and/or made a bad diagnosis. The iOSCE is an innovative approach to better reveal the various problems of learning and performance students encounter during rotations while hastening the learning and improvement in performance through a safe medical environment.

Although the type and clarity of instructions and level of complexity of tasks varied from one station to the next, the iOSCE methodology received very satisfactory comments from all participants. Participants expressed that the iOSCE experience at Taipei Medical University was better than the OSCE previously conducted in that it clarified what was to be assessed as well as allowing the participant to demonstrate his/her diagnostic reasoning better writing a simple patient note. The candidate was now able to show how he/she could integrate the information obtained from the SP with the proper diagnostic blood and imagine studies from the VP to develop a realistic differential diagnosis and plan.

Overall the iOSCE concept or methodology has the following advantages:

1) iOSCE uses all qualified cases, all coming from real patients.

2) Educators can get the information from the DxR© Clinician easily to make up a scenario to be used with an SP.

3) The faculty can get real time students performance and give them immediate feedback for proper learning and personalized teaching since the utility record provides deficient and incomplete information.

4) The students experience the process of making a real diagnosis.

### Limitations

Our study was limited by relatively small numbers of participants and a limited number of cases. We plan to design a study with more examinees and more cases so we can further compare the OSCE and iOSCE advantages and disadvantages.

## Conclusion

Using the iOSCE methodology to evaluate competency is an effective method as shown by the results in this study and by the feedback obtained from students. The following observations were made by teaching staff/examiners while students/examinees underwent iOSCE: examiners can get a real time feedback, find student learning deficiencies and help students learn from their mistakes. Aided by the implementation of iOSCE, teaching staff can better see where students are deficient and can easier set up case learning environments to hasten the student learning process. However, further design and experimentation must be done before such methodology can be considered for a possible standard exam.

## Competing interests

The authors declare that they have no competing interests.

## Authors’ contributions

CWL has developed the iOSCE methodology, supervised the study, acquired necessary data and has critically revised the manuscript. DLC has organized, analyzed and interpreted the data and drafted the entire manuscript. MHS and GSL critically analyzed the data and wrote parts of the manuscript. CCW, CYC and FPL have organized and facilitated the study, interviewed and selected participants, helped organize the iOSCE program and have provided critical analysis of this study. YCL helped with critical analysis and interpretation of data and helped organize, facilitate and supervise this study. All authors read and approved the final manuscript.

## Pre-publication history

The pre-publication history for this paper can be accessed here:

http://www.biomedcentral.com/1472-6920/13/102/prepub
